# Effect of Mistuning on the Detection of a Tone Masked by a Harmonic Tone Complex

**DOI:** 10.1371/journal.pone.0048419

**Published:** 2012-11-05

**Authors:** Martin Klein-Hennig, Mathias Dietz, Astrid Klinge-Strahl, Georg Klump, Volker Hohmann

**Affiliations:** 1 Medical Physics, Carl-von-Ossietzky-Universität Oldenburg, Oldenburg, Germany; 2 Animal Physiology and Behavior, Carl-von-Ossietzky-Universität Oldenburg, Oldenburg, Germany; University of Salamanca- Institute for Neuroscience of Castille and Leon and Medical School, Spain

## Abstract

The human auditory system is sensitive in detecting “mistuned” components in a harmonic complex, which do not match the frequency pattern defined by the fundamental frequency of the complex. Depending on the frequency configuration, the mistuned component may be perceptually segregated from the complex and may be heard as a separate tone. In the context of a masking experiment, mistuning a single component decreases its masked threshold. In this study we propose to quantify the ability to detect a single component for fixed amounts of mistuning by adaptively varying its level. This method produces masking release by mistuning that can be compared to other masking release effects. Detection thresholds were obtained for various frequency configurations where the target component was resolved or unresolved in the auditory system. The results from 6 normal-hearing listeners show a significant decrease of masked thresholds between harmonic and mistuned conditions in all configurations and provide evidence for the employment of different detection strategies for resolved and unresolved components. The data suggest that across-frequency processing is involved in the release from masking. The results emphasize the ability of this method to assess integrative aspects of pitch and harmonicity perception.

## Introduction

The harmonicity of a signal is an important feature in auditory grouping. It allows humans to group frequency components that have a common fundamental frequency (F0) into a single auditory object (e.g., [1]). This helps, for example, in the segregation of concurrent speech from different talkers or speech from noise (e.g., [2]).

In a harmonic complex it is difficult to detect or “hear out” single frequency components. To facilitate this task, additional cues are needed. The most commonly used cue is mistuning, i.e., shifting the frequency of a target component in such a way that it no longer matches the harmonic frequency pattern defined by the F0 of the complex (e.g., [3]–[6]). Mistuning effectively provides release from masking, enabling or facilitating the detection of a single component that would otherwise be masked by the rest of the harmonic complex. This masking release effect has been shown only indirectly in studies on mistuning detection (e.g. [3], [5]), as they measured the just noticeable amount of mistuning using a paradigm in which the subjects compared mistuned complexes to harmonic complexes, while the amount of mistuning was adaptively varied. A direct investigation of the effect of mistuning on detection thresholds is possible in detection experiments, where a single component of a complex is the target signal that has to be detected, and the rest of the complex is regarded as the masker, effectively masking the component ([7], [8]). In these experiments, the level of the target component is varied adaptively to obtain detection thresholds for various stimulus configurations. This method has the advantage that it generates detection thresholds that allow comparison to and possibly combination with other masking release effects such as comodulation masking release (e.g., [9]) or binaural unmasking (e.g., [10]), as these effects are also investigated by measuring and comparing detection thresholds. The results of the mistuning detection studies mentioned above cannot be expressed in terms of masking level differences, rendering the comparison to other masking release effects difficult. The two studies that investigated single-component detection in harmonic complexes ([7], [8]) have several methodological specifics and limitations that make it difficult to derive a comprehensive picture of the auditory processing involved. Oh and Lutfi [7] used non-deterministic frequency configurations in their stimuli, as they designed their experiment in the context of informational masking, whereas Klinge et al. [8] used deterministic frequency configurations. Klinge et al. [8] performed their measurements in free-field, which makes control of the presented stimuli at the ear-level difficult. Both studies measured detection thresholds for one fixed percentage of mistuning only.

To shed further light on the effects involved in harmonicity processing, this study provides a data set of detection thresholds for a single sinusoidal target tone masked by harmonic and mistuned complexes for an extensive set of critical conditions. The stimuli consisted of a harmonic complex as masker and a single sinusoidal target component that was either harmonic or mistuned to the masker’s fundamental frequency. The stimuli were presented in a controlled environment with headphones, with an adaptively varied target level. In order to test the ability of the method to account for different strategies to detect the mistuned component, thresholds were obtained for frequency configurations in which the harmonics of the tone complex are either resolved or unresolved. The possible involvement of across-frequency processes in the detection of a mistuned component is investigated by increasing the stimulus bandwidth.

## Materials and Methods

### Ethics Statement

Written consent was obtained from each participant prior to the experiments. The experiments were approved by the local ethics committee of the University of Oldenburg.

### Subjects

Six normal-hearing listeners (3 male, 3 female), aged 22 to 27, took part in the study. Pure tone audiograms were measured for all test subjects, showing no hearing loss (>15 dB HL) between 250 and 8000 Hz. Prior to data collection, all subjects completed a 3-hour training run with the same stimuli as used in the experiments.

### Stimuli

**Figure 1:**
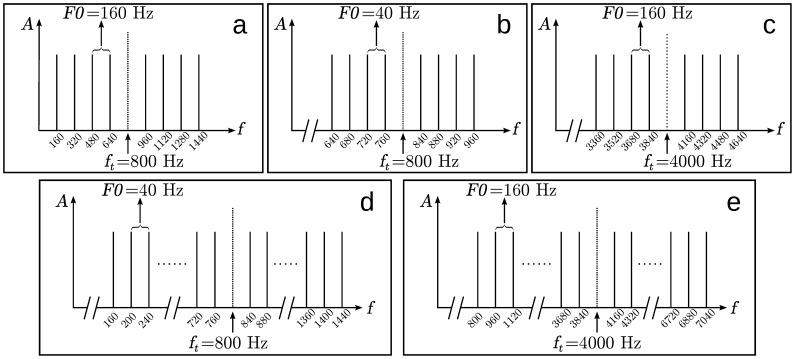
Pictogram of the spectra of the stimuli used in the experiments. Black lines indicate the harmonics of the tone complex used as masker. The harmonics are multiples of the masker’s fundamental frequency F0. The lowest harmonic is not necessarily the fundamental frequency. The dotted line shows the frequency 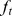 of the target component that had to be detected and was not part of the masker. Panels a to c show the frequency configurations of Experiments 1–3 in the harmonic condition. Panels d and e show the frequency configurations of the broad-band conditions with additional masker harmonics as used in Experiments 2 and 3.

The stimuli consisted of a harmonic complex used as masker and a pure tone target signal. The masker was generated by adding up eight pure tones of different frequencies in random phase for each stimulus presentation. Randomization of the phases was applied to prevent subjects from learning spectral or temporal templates and exploiting them in the detection task. The frequencies were integer multiples of the fundamental frequency F0 of the masker and were the four harmonics below and above the target signal frequency 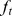. In Experiment 1, a fundamental frequency F0 = 160 Hz was used, with a target frequency of 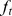 = 800 Hz (see 1a). To generate unresolved harmonics in Experiment 2, F0 was set to 40 Hz, while keeping 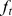 at 800 Hz (see Fig. 1b). Experiment 3 also had unresolved harmonics in a high frequency range, by shifting 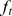 to 4 kHz, while keeping F0 at 160 Hz (see Fig. 1c). A harmonic is defined as resolved if it individually excites a place on the basilar membrane (e.g., [11]), i.e., it is the only harmonic that occurs within the equivalent rectangular bandwidth (ERB, see [12]) of an auditory filter centered around that harmonic’s frequency. If multiple harmonics fall into the same ERB, they are defined as being unresolved.

Additionally, Experiments 2 and 3 contain broad-band conditions that were configured to have the same bandwidth-to-target-frequency ratio as Experiment 1. This was achieved by additional masker components below and above the target, leading to stimuli with 32 and 40 components in Experiments 2 and 3, respectively (see Figures 1d and 1e).

Each of the masker components had a level of 55 dB SPL, resulting in an overall masker level of 64 dB SPL. The broad-band conditions of Experiments 2 and 3 had overall masker levels of 70 and 71 dB SPL, respectively. Target and masker were gated and presented synchronously with 25 ms Hanning windows. The total stimulus duration was 400 ms.

In order to create a mistuned condition, the F0 of the masker was increased while keeping the frequency of the target 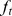 constant, which effectively led to a downward mistuning of the target component. To have a comparable amount of mistuning in all experiments, the percentage of mistuning was chosen such that the fourth masker component, which is next to the target component, was shifted upwards by 10, 20 and 40 Hz in Experiments 1 and 2, as these values are proper divisors of the F0s of the stimuli. Higher frequency shifts of 20, 40, 80 and 160 Hz were used in Experiment 3 due to the high target frequency of 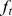 = 4 kHz. The fourth component was selected as a measure of mistuning since it is the most likely component to fall into the auditory filter centered on the target frequency after mistuning.

### Procedure

The experiments were conducted in a double-walled, sound-attenuating booth. The stimuli were generated digitally with a sampling frequency of 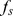 = 48 kHz and presented via Sennheiser (Wedemark-Wennebostel, Germany) HD 650 headphones. The headphone was free-field calibrated on a Brüel&Kjæ r (Næ rum, Denmark) 4135 artificial ear. The subjects responded using a computer keyboard and visual feedback was provided on a computer monitor.

A 3-interval 2-alternative forced-choice procedure was used to measure the detection thresholds. Using a 1-up 2-down tracking rule, the 70.7%-correct point on the psychometric function was estimated (see [13]). The reference intervals did not contain the target signal. The first interval presented to the subjects was always a reference interval, and could not be selected. The target signal was first presented with a level of 65 dB SPL, which was initially varied in 5 dB steps. Stepsize was reduced to 2 and 1 dB after the second and fourth reversal, respectively.

Each adaptive run was terminated after eight reversals with 1 dB steps. The individual means were obtained by averaging over the last eight reversals of five experimental runs. The mean thresholds were obtained by averaging over the individual means of the subjects.

## Results

### Experiment 1: Resolved Harmonics, F0 = 160 Hz

**Figure 2:**
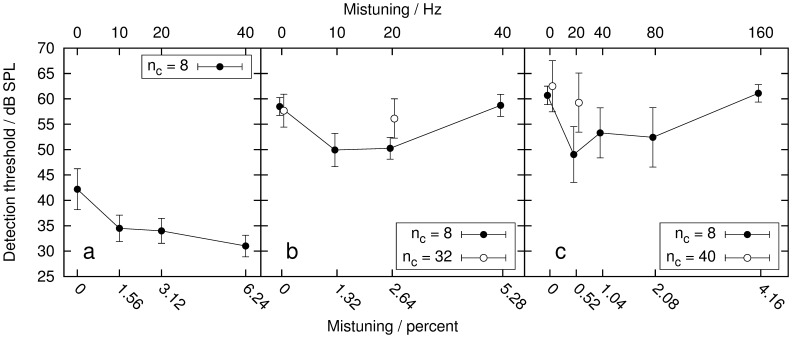
Detection thresholds of the single target component in dB SPL as a function of mistuning in percent (bottom axis) or in Hz (top axis). Filled circles indicate thresholds obtained with a masker comprised of eight components (i.e. 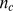 = 8). Open circles show the thresholds obtained with the broadband conditions, with 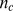 = 32 in Experiment 2, and 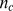 = 40 in Experiment 3. Panel a: Thresholds obtained in Experiment 1, with resolved harmonics, with a fundamental frequency F0 = 160 Hz and a target frequency 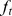 = 800 Hz. Panel b: Thresholds obtained in Experiment 2, with unresolved harmonics, with a fundamental frequency F0 = 40 Hz and a target frequency 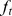 = 800 Hz. Panel c: Thresholds obtained in Experiment 3, with unresolved harmonics, with a fundamental frequency F0 = 160 Hz and a target frequency 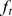 = 4000 Hz. The error bars indicate the standard deviation across six normal-hearing subjects.

In this experiment, the masker had a fundamental frequency of 160 Hz and the target signal frequency was 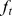 = 800 Hz (5^th^ harmonic, see Fig. 1a). This way, all harmonics were resolved. The results are shown in Fig. 2a.

With increasing mistuning, the masked threshold decreased from 42 dB SPL to 31 dB SPL. Thus, an 11 dB release from masking was found. A repeated-measures analysis of variance (ANOVA) showed a highly significant main effect of mistuning on the detection threshold: F(3,15) = 28.7, p<0.001. Post-hoc pairwise comparisons (Bonferroni corrected) indicated that the harmonic 0.0% condition was significantly different from the mistuned conditions (p<0.001). The mistuned conditions were not significantly different from each other (assuming 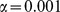).

### Experiment 2: Unresolved Harmonics, F0 = 40 Hz

In Experiment 2, F0 was set to 40 Hz, whereas 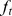 was kept at 800 Hz (20th harmonic, see Fig. 1b). With these settings, the components of the complex were unresolved. The results are shown in Fig. 2b. As in Experiment 1, a threshold decrease with increasing mistuning can be observed in the first three conditions with 8 masker harmonics. The 0.0% condition as well as the 5.28% condition yield a threshold of 58 dB SPL, since a mistuning value of 5.28% creates a harmonic condition similar to the 0.0% condition, as the frequency of the fourth masker component is shifted from 760 to 800 Hz. Thus, the frequency of the fourth component coincides with the target frequency. For the 1.32% and 2.64% conditions, thresholds of 50 dB SPL are found, leading to a maximal difference in masked threshold of 8 dB. A repeated-measures ANOVA showed a highly significant main effect of mistuning on the detection threshold: F(3,15) = 46, p<0.001. Post-hoc pairwise comparisons (Bonferroni corrected) indicated that the thresholds of the 0.0% condition and the 5.28% condition were significantly different from the thresholds in the other conditions (p<0.001). There was no significant difference between the thresholds of the 1.32% and 2.64% mistuned conditions and no significant difference between the 0.0% and 5.28% condition.

For the broad-band condition, the thresholds for 0.0% and 2.64% mistuning were 58 and 56 dB. In this condition there was no significant effect of mistuning on the thresholds: F(1,5) = 2.51, p = 0.17. Comparing the thresholds of the 2.64% conditions with 8 and 32 masker harmonics, it was found that the inclusion of additional harmonics in the masker had a significant effect on the mistuned thresholds: F(1,5) = 26.96, p<0.01. The thresholds of the 0.0% condition were not significantly influenced by the increase of masker bandwidth: F(1,5) = 0.35, p = 0.58.

### Experiment 3: Unresolved Harmonics, F0 = 160 Hz

Here, the masker fundamental frequency was again set to F0 = 160 Hz, but the target frequency was 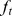 = 4 kHz Hz (25^th^ harmonic, see Fig. 1c). In addition to the harmonic masker and the target signal, a continuous white noise, second-order low-pass filtered (butterworth) at 1.5-kHz, was presented throughout the experiment to interfere with low-frequency distortion products that could influence the detection. The noise had an overall level of 40 dB SPL. In Fig. 2c, the results show a decrease in masked thresholds with increasing mistuning between the first two conditions with 8 masker harmonics. The maximal masking release of 11 dB can be found between the 0.0% and 0.52% conditions. The 0.0% and 4.16% conditions yield the same threshold, as the mistuning value of 4.16% creates a harmonic condition similar to the 0.0% condition, as in Experiment 2. A repeated-measures ANOVA showed a highly significant main effect of mistuning on the detection threshold: F(4,20) = 15.49, p<0.001. Post-hoc pairwise comparisons (Bonferroni corrected) indicated that the 0.0% condition and the 4.16% condition were significantly different from the other conditions (p<0.05). There was no significant difference between the 0.52%, 1.04% and 2.08% mistuned conditions, and no significant difference between the 0.0% and 4.16% condition (assuming 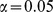).

For the broad-band condition, the thresholds for 0% and 0.52% mistuning were obtained. The detection threshold decreased from 62 to 59 dB when a mistuning of 0.52% was applied. The effect of mistuning on these two thresholds was significant: F(1,5) = 20, p<0.01. Comparing the 0.52% conditions with 8 and 40 masker harmonics, it can be seen that the inclusion of additional harmonics in the masker had a significant effect on the thresholds: F(1,5) = 23.32, p<0.01. The thresholds of the 0.0% conditions with 8 and 40 maskers were not significantly influenced by the increase of masker bandwidth: F(1,5) = 1.4, p = 0.28.

## Discussion

The method of measuring detection thresholds of a single component masked by a harmonic complex yielded reliable and statistically significant results that are in line with previously published data. A significant effect of mistuning on the masked threshold of the target component has been shown in all three experiments. Comparing the harmonic condition to the mistuned condition with the lowest threshold, the results show release from masking between 8 and 12 dB. This outcome is in line with [7] and [8]. In a comparable condition with a target frequency of 1 kHz and 10 masker components, Oh and Lutfi [7] measured a masking release of about 5 dB between harmonic and mistuned stimuli. The smaller amount of masking release in that study could be caused by the nondeterministic stimuli, which consisted of 10 randomly distributed components between 200 Hz and 10 kHz. Klinge et al. [8] observed masking release of about 7 dB with a target frequency of 1 kHz, and 11 dB with a target frequency of 8 kHz.

In Experiments 2 and 3, where the harmonics were unresolved, detection thresholds of both harmonic and mistuned conditions were increased by up to 18 dB compared to Experiment 1. This is similar to [8], where an increase of 11 to 14 dB was observed comparing the thresholds of a 1-kHz target and an 8-kHz target. The increased thresholds occur due to the unresolvability of the target component. Additional masker energy in the target frequency filter, which is present in the unresolved conditions as multiple components falling into the same cochlear filter, decreases the signal-to-masker energy ratio and makes it more difficult to detect the target.

In Experiment 1, where the harmonics are resolved, the masking release by mistuning could occur due to auditory object separation. As the target component is mistuned relative to the masker fundamental frequency, it is not fused with the masker harmonics into a single auditory object, but rather stands out and is perceived as a separate tone. This was also reported by the test subjects in [3].

However, a large effect of mistuning was also found in the unresolved conditions in this study and in [8]. In these conditions, object separation might play a role, but target detection could also be based on further signal features that were not present in the resolved condition. Klinge et al. [8] hypothesized that the predominant cue in their unresolved condition was a change of the temporal envelope of the stimulus waveform caused by adding the target signal to the complex masker. As their harmonic complexes were generated by adding up pure tones in sine phase, their stimuli had a constant envelope waveform throughout the experiment. In this study, the phases of the components were randomized in each presentation interval, and a large release from masking could still be found. This observation is incompatible with a detection mechanism based on a stored temporal template of the stimuli, as discussed by Klinge et al. [8]. In [3], the subjects reported that they could only hear out mistuned lower harmonics. They were not able to hear out higher (i.e. unresolved) harmonics and hence used a different mechanism for detecting the mistuned interval. They reported that the mistuning produced beats that allowed to identify the mistuned interval in the task. These beats are presumed to be produced by either combination tones (e.g. [14]) or a change in phase relation between the harmonics produced by mistuning. The phase-relation hypothesis is not compatible with the results of this study, as mentioned above. A second hypothesis is that combination tones or distortion products generated in the cochlea were the cues that allowed identifying the target interval, since the reference and target intervals differ in their combination tone patterns. The measured thresholds would then reflect the level at which these combination tones can no longer be perceived. In the harmonic conditions, a detection mechanism based on combination tones cannot be used, as the target and reference intervals have the identical combination tone frequencies. This explains the high thresholds in the harmonic unresolved conditions. As a low-pass noise was used in Experiment 3 to mask possible distortion products, this detection mechanism is either not used in Experiment 3, or the noise level of 40 dB SPL was not sufficient for masking the distortion products. In a supplementary experiment with only one test subject and 4 runs per condition, a noise of 50 dB SPL was used and Experiment 3 was repeated for the 0.0% and 0.52% conditions. The test subject was still able to achieve a masking release by mistuning of 10 dB. This suggests that the distortion components were effectively masked at 40 dB SPL. It is possible that a combination of two possible detection mechanisms, i.e., masking release due to object separation and due to the detection of combination tones is used.

Within each experiment, the mistuned thresholds were not significantly different from each other (except for the 5.28% condition in Exp. 2 and the 4.16% condition in Exp. 3). The smallest amount of mistuning already produced the largest amount of masking release. In Experiment 1, the smallest mistuning of 1.56% is above the mistuning detection thresholds observed in [3] for similar frequency configurations. They found a mistuning detection threshold of 0.94% for the 800-Hz component in a harmonic complex with F0 = 200 Hz. All other mistuning values in Experiment 1 are above this threshold. Therefore, no effect of the mistuning detection threshold as such is to be expected. The reason for the slightly increasing masking with increasing mistuning (i.e. higher detection thresholds compared to the condition with the lowest mistuning) might be a combination of two effects. With increasing mistuning, masking release from object separation increases and energetic masking increases, because the target and one of the masker components move closer in frequency. These effects could both be too small to have a significant effect on the thresholds, or both effects might cancel each other out, either way resulting in the observed flat response. For harmonic numbers greater than five, [3] report decreasing mistuning detection thresholds with increasing harmonic number, with thresholds as low as 0.2% for the 2.4-kHz component in a 200-Hz complex. Thus, all mistuning values in Experiments 2 and 3 are also above the mistuning detection threshold and the same argument as for Experiment 1 applies.

While in the narrow-band conditions an effect of mistuning on the detection threshold was observed, this effect disappeared in the broadband conditions, indicating that signal components outside the auditory filter of the target can play a role. Increasing the number of remote masker components does not change the internal signal in the on-frequency channel around the target component. Thus, a single-channel auditory model would not be sufficient to predict the decrease of masking release in the broadband conditions. This points towards the involvement of across-frequency processes. One possible candidate for this across frequency process is the pitch strength of the additional lower harmonics that were added to the complex to increase bandwidth. Pitch perception is dominated by low-frequency resolved harmonics (e.g., [5]), thus adding low-frequency components to the masker, as done in Experiments 2 and 3 by adding components from the fourth harmonic upwards, creates a strong cue for the masker F0, that might hamper the detection of the mistuned target tone. According to this hypothesis, object binding would be stronger for increased pitch strength and could be responsible for the decreasing masking release. This would be in line with [15], who found that F0 discrimination performance decreased with increasing lowest harmonic number of a tone complex.

### Conclusions

We presented a method to investigate the influence of mistuning on component detection by measuring release from masking of a single target component in harmonic complexes as a function of mistuning. The results show that the method is able to assess effects in harmonicity and pitch perception and can account for effects of resolvability. The measured detection thresholds allow for quantification of the masking release effect by mistuning and challenge current auditory processing models, in particular because of the observed across-frequency interaction.
